# Growth and Strain Modulation of GeSn Alloys for Photonic and Electronic Applications

**DOI:** 10.3390/nano12060981

**Published:** 2022-03-16

**Authors:** Zhenzhen Kong, Guilei Wang, Renrong Liang, Jiale Su, Meng Xun, Yuanhao Miao, Shihai Gu, Junjie Li, Kaihua Cao, Hongxiao Lin, Ben Li, Yuhui Ren, Junfeng Li, Jun Xu, Henry H. Radamson

**Affiliations:** 1 Key Laboratory of Microelectronic Devices & Integrated Technology, Institute of Microelectronics, Chinese Academy of Sciences, Beijing 100029, China; wangguilei@ime.ac.cn (G.W.); sujiale@ime.ac.cn (J.S.); miaoyuanhao@ime.ac.cn (Y.M.); lijunjie@ime.ac.cn (J.L.); renyuhui@ime.ac.cn (Y.R.); lijunfeng@ime.ac.cn (J.L.); 2 School of Integrated Circuits, University of Chinese Academy of Sciences, Beijing 100049, China; 3 Beijing Superstring Academy of Memory Technology, Beijing 100176, China; 4 School of Integrated Circuits, Tsinghua University, Beijing 100086, China; jun-xu@tsinghua.edu.cn; 5 Research and Development Center of Optoelectronic Hybrid IC, Guangdong Greater Bay Area Institute of Integrated Circuit and System, Guangzhou 510535, China; linhongxiao@ime.ac.cn (H.L.); liben@ime.ac.cn (B.L.); 6 Research and Development Center of High Frequency and High Voltage Devices and Integration, Institute of Microelectronics, Chinese Academy of Sciences, Beijing 100029, China; xunmeng@ime.ac.cn; 7 NAURA Technology Group Co., Ltd., Beijing 100176, China; gushihai@naura.com; 8 Fert Beijing Institute, School of Integrated Science and Engineering, Beihang University, Beijing 100191, China; kaihua.cao@buaa.edu.cn; 9 Department of Electronics Design, Mid Sweden University, Holmgatan 10, 85170 Sundsvall, Sweden

**Keywords:** GeSn growth, selective etch, strain modulation, RPCVD

## Abstract

GeSn materials have attracted considerable attention for their tunable band structures and high carrier mobilities, which serve well for future photonic and electronic applications. This research presents a novel method to incorporate Sn content as high as 18% into GeSn layers grown at 285–320 °C by using SnCl_4_ and GeH_4_ precursors. A series of characterizations were performed to study the material quality, strain, surface roughness, and optical properties of GeSn layers. The Sn content could be calculated using lattice mismatch parameters provided by X-ray analysis. The strain in GeSn layers was modulated from fully strained to partially strained by etching Ge buffer into Ge/GeSn heterostructures . In this study, two categories of samples were prepared when the Ge buffer was either laterally etched onto Si wafers, or vertically etched Ge/GeSnOI wafers which bonded to the oxide. In the latter case, the Ge buffer was initially etched step-by-step for the strain relaxation study. Meanwhile, the Ge/GeSn heterostructure in the first group of samples was patterned into the form of micro-disks. The Ge buffer was selectively etched by using a CF_4_/O_2_ gas mixture using a plasma etch tool. Fully or partially relaxed GeSn micro-disks showed photoluminescence (PL) at room temperature. PL results showed that red-shift was clearly observed from the GeSn micro-disk structure, indicating that the compressive strain in the as-grown GeSn material was partially released. Our results pave the path for the growth of high quality GeSn layers with high Sn content, in addition to methods for modulating the strain for lasing and detection of short-wavelength infrared at room temperature.

## 1. Introduction

GeSn has aroused extensive attention as a result of its direct bandgap properties [1], compatibility with Si CMOS processes [2,3,4,5,6], higher absorption coefficients at short-wavelength infrared (SWIR) windows [7], and higher carrier mobilities compared with Si and Ge, etc. [3]. These characteristics show that GeSn materials will be promising for both optoelectronics and high-speed electronics for silicon photonic-electronic platforms in the near future. It is also expected that GeSn could open applications for thermoelectric materials in the near future [8,9]. There are several challenges in growing high quality GeSn: (I) the lattice mismatch between Ge and Sn is 14.7% and is even higher at 17% between Sn and Si; (II) incorporation of Sn in Ge is difficult due to the low solubility (<1%) of Sn in Ge and the instability of α-Sn above 13 °C; and (III) Sn precipitation and Sn agglomeration occur during growth. Therefore, growth tools, such as molecular beam epitaxy (MBE) [10,11,12], reduced pressure chemical vapor deposition (RPCVD) [13,14,15,16,17,18,19,20,21,22], ultra vacuum chemical vapor deposition (UHVCVD) [23], physical vapor deposition (PVD) [24], and sputtering technique [25,26] have been proposed to grow GeSn at low temperatures. Compared to MBE and PVD, CVD has the advantages of lower costs, higher growth rates, larger wafer sizes, and mass production potential which can easily be transferred to the Si-based microelectronic and photonic industry. As early as 2001, Kouvetakis’s group from Arizona State University (ASU) grew GeSn and GeSnSi via reactions of Si–Ge hydrides and SnD_4_ in a UHVCVD chamber, but SnD_4_ molecules are very unstable [27,28]. Then, in 2011, researchers from IMEC [14] and KTH proposed the growth of GeSn using commercially available reaction precursors (SnCl_4_/Ge_2_H_6_) in a production RPCVD tool [18]. Since then, many research groups reported the growth of GeSn using precursor combinations of SnCl_4_/Ge_2_H_6_ and SnCl_4_/GeH_4_ [13,14,15,16,17,18,19,20,21,22,23]. Moreover, GeSnSiC alloys were also grown using Si_2_H_6_, Ge_2_H_6_, SnCl_4_, and SiCH_6_, precursors which are also important for photonic applications [19,20]. From the mass production perspective, GeH_4_ is preferred to Ge_2_H_6_, owing to its wider usage and lower cost. In order to improve material properties, the effects of growth temperature, growth pressure, carrier gas, strain relaxation, and doping on GeSn growth were systematically investigated. Experimental results indicate that growth temperature, growth pressure, and strain relaxation were major factors towards achieving high Sn composition and high quality GeSn [29,30].

With the rapid development of GeSn CVD growth techniques, truly direct-bandgap transition GeSn material was experimentally verified and single PL peaks with narrow line-widths at each temperature were clearly observed [31]. Inspired by this research, the first optically pumped FP cavity GeSn laser was demonstrated at low temperature [32]. From then on, other lasers were successfully demonstrated: GeSn micro-disk lasers [33], 2D hexagonal photonic crystal (PC) cavity GeSn lasers [34,35], micro-bridge cavity GeSn lasers [36], 1D PC cavity GeSnOI lasers [37], GeSn/SiGeSn quantum wells lasers [38], GeSn micro-disk continuous wave (CW) lasers [39,40] and electrically pumped GeSn/SiGeSn lasers [41,42]. However, their thresholds were still very high, necessitating strategies to achieve both room temperature lasing and low thresholds. The main issue for room temperature GeSn lasing is the presence of compressive strain in GeSn/Ge, which will reduce its directness in the bandgap. In the meantime, it is also highly desirable that Sn distribution in the GeSn optical gain medium be uniform. In order to obtain good device performance, the processes of making device structures, especially the etching process, become very important. A good etching process can not only obtain a high selection ratio between different materials, but can also have very important effects on the interface state and subsequent alloy processes.

GeSn thin films on Ge are subjected to compression strain which still result in indirect bandgaps. When the composition of Sn is more than the critical value of 6–8% GeSn will realize direct bandgap transition. One way to overcome the compression strain of Ge_1−x_Sn_x_ thin films is to selectively remove the stress-induced Ge buffer layer [43,44,45,46,47]. This method can be used to prepare strain-free, direct bandgap Ge_1−x_Sn_x_. Y. Han et al. used wet etching and obtained a selective etching ratio of 8:1 [46]; they also found that when the Sn concentration is above 6%, selectivity increases as much as 336. CF_4_ is used as dry etching gas for the Ge buffer, and to make Ge_0.92_Sn_0.08_ micro-disks [43,44,45,46,47,48]. A. Campo et al. reported that when adding 30% O_2_ in CF_4_, Ge etching has the highest etching rate and has the highest selectivity with Si, since the Si etch rate is controlled by the thickness of the SiO_x_F_y_ superficial layer; in contrast, the thickness of GeO_x_F_y_ does not inhibit Ge etching [49]. Although a series of studies have demonstrated GeSn growth and good performance devices, achieving GeSn growth with high Sn content and high epitaxial quality are still challenges due to Sn segregation. New methods are still being sought to deal with incorporation of high Sn content in Ge with low defect densities. 

In comparison to GeSn/Ge, a better candidate for optoelectronic applications is GeSn-on-insulator (GeSnOI). This is a result of the excellent mobility of GeSnOI, in addition to its higher light emission efficiency, higher net gain, great optical confinement, low leakage current, resonator effect, higher operation temperature, lower coupling loss with waveguide, and its greater ease for photonic integration [4,50,51]. 

This paper presents a novel method to grow GeSn layers on Ge buffer with high Sn content and high crystalline quality. We present an X-ray diffraction method to precisely determine the Sn content in GeSn. In comparison with the classical way of using Rutherford backscattering spectrometry (RBS), our method provides a more cost-effective and faster analysis. The strain amount in GeSn could be modulated by vertical or lateral etching of Ge buffer in micro-disks. In this process, the GeSn/Ge (virtual substrate) layers are transferred to form GeSnOI wafers, and the top Ge layer on GeSnOI has great influence on strain in GeSn. The strain relaxation of GeSn was obtained by under-etching with Ge buffer in the GeSn/Ge heterostructure in micro-disk arrays formed on Si wafers, or back-etching during the formation of GeSnOI wafers. The high growth rate and low defect density of the GeSn layer provide PL at room temperature, which offers the unique possibility of depositing multilayer structures for lasers and detectors operating in the SWIR region. In this study, both selective dry and wet etching methods were investigated. We present two kinds of process flow, and obtained two kinds of micro-disks: partial strain release and complete strain release. Micro-disk morphology and strain release of the different selective etching methods were also measured using an X-ray technique in global characterization and using transmission electron microscopy (TEM), as well as nanoelectron beam diffraction (NBD) in local analyses. 

## 2. Materials and Methods

**Figure 1:**
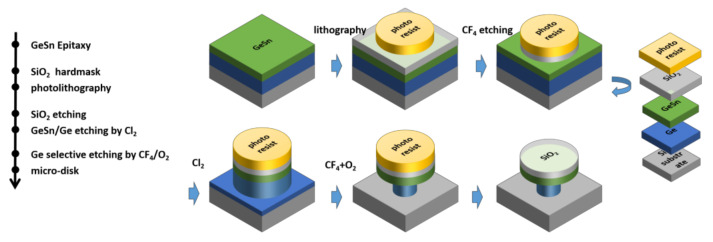
Formation of partial strain-relaxed GeSn micro-disk with SiO_2_ as hard mask: process flow and schematic of the process.

GeSn samples were grown on Si (100) wafers by applying SnCl_4_ and Ge_2_H_6_ as precursors at 285–320 °C in a commercial RPCVD tool (Epsilon 2000, ASM). High-equality Ge buffer layers of one micrometer were grown prior to the deposition of GeSn layers. In this study, strain in the GeSn layer was modulated either through lateral etching of the Ge buffer in the micro-disks of Ge/GeSn formed on the Si bulk wafer, or vertical etching of Ge on the Ge/GeSnOI wafer. Figure 1a shows the process flow of forming micro-disks, starting with deposition of a 100-nanometer SiO_2_ film with PECVD serving as a hard mask on Ge/GeSn heterostructures . Circular disks with diameters of 3 nm, 6 nm, and 9 nm were patterned. The GeSn/Ge heterostructure was vertically etched using Cl_2_ precursor while the GeSn layer was selectively etched using CF_4_/O_2_ plasma gas, as shown in Figure 1b. Another group of micro-disks were formed with no SiO_2_. In order to ensure that lateral selective etching is not out of the way, during the first step of etching it was not etched directly reaching the Si layer, but retain a portion of the Ge. Then, CF_4_ and O_2_ were used for selective etching. After several repeated experiments, an ultra-thin strain, relaxed GeSn micro-disk structure was obtained. The etching was carried out using an inductively coupled plasma (ICP) PlasmaPro^®^ 100 Cobra with frequency 13.56 MHz and a background vacuum of less than 4 × 10^−6^ Torr. CF_4_ was used as the etching gas to etch SiO_2_. The dry etching process adopted CF_4_, the air pressure was controlled at 5 mT, the reaction temperature of the mixed gas was 20 °C, the upper RF power used was 400 W, the lower RF power was 100 W, and the total flow rate of CF_4_ was 50 sccm. Then, the GeSn and Ge layers were vertically etched downward by chlorine (Cl_2_). The air pressure was controlled at 5 mT, the reaction temperature of the mixed gas was 20 °C, the upper RF power used was 300 W, the lower RF power was 45 W, and the total flow rate of Cl_2_ was 35 sccm. The GeSn micro-disk structure was obtained by transverse selective etching of Ge by CF_4_ and O_2_. The pressure was controlled at 90 mT, the reaction temperature of the mixed gas was 20 °C, the upper RF power used was 200 W, the lower RF power was 0 W, and the total flow rate of CF_4_/O_2_ was 100 sccm. In terms of the volume percentage of CF_4_/O_2_, the volume ratio of CF_4_ was 70%, and the volume ratio of O_2_ was 30%. We reduced the RF power to 0 W to eliminate surface damage caused by ion bombardment. The GeSn film is thin, and this setting protected the morphology of GeSn from being damaged by particle bombardment. The decrease in RF power was accompanied by a corresponding decrease in the reactive free radical concentration and resulted in a slower etching rate. The addition of 30% oxygen increased the etching rate of Ge to its highest, thus even when RF was reduced to 0 W, Ge still had an etching rate of 200 nm/min.

**Figure 2:**
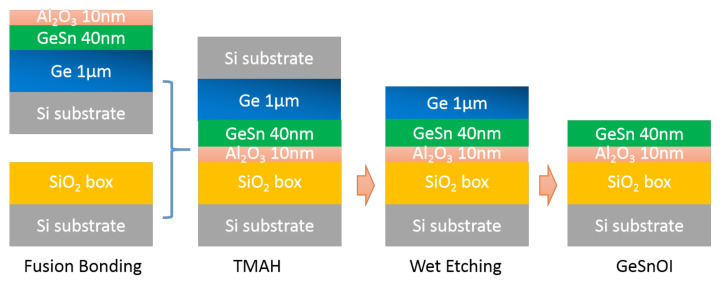
Process flow for manufacturing of GeSnOI substrates.

GeSnOI wafers were processed when the GeSn/Ge heterostructure with a 10-nanometer Al_2_O_3_ cap layer could be bonded to 520 nm SiO_2_ on a support wafer by fusion bonding. The Si substrate was back polished and completely removed by TMAH to the Ge layer. Later, strain in the GeSn layer could be modulated by stepwise etching of the Ge buffer using NH_3_OH: H_2_O_2_: H_2_O (1:4:25) solution; this process is shown in Figure 2. The etching times were 20 s, 100 s, and 120 s, where the 500 nm, 100 nm, and 20 nm Ge buffer layers could finally remain on the GeSn layers, respectively. Detailed information of the etching process is shown in Table 1.

High-resolution X-ray diffraction (HR-XRD), Rutherford backscattering spectrometry (RBS), atomic force microscopy (AFM), transmission electron microscopy (TEM), and photoluminescence (PL) spectra were used to characterize the structural and optical properties for the as-grown GeSn samples, GeSn micro-disk structures, and GeSnOI wafers.

## 3. Results and Discussion

### 3.1. Growth Kinetics

The fundamental problem with growing GeSn layers is segregation of Sn atoms. There are two main factors which govern Sn segregation: chemical and size effects. The first effect depends on the heats of sublimation of Sn (65 kcal/mole) and Ge (89 kcal/mole), which in fact determine whether Sn-to-Sn or Sn-to-Ge bonding is favored. The size effect originates from the size difference between Sn and Ge atoms. Therefore, we may conclude that high incorporation of Sn in Ge is a real challenge as a result of both aforementioned effects. 

**Figure 3:**
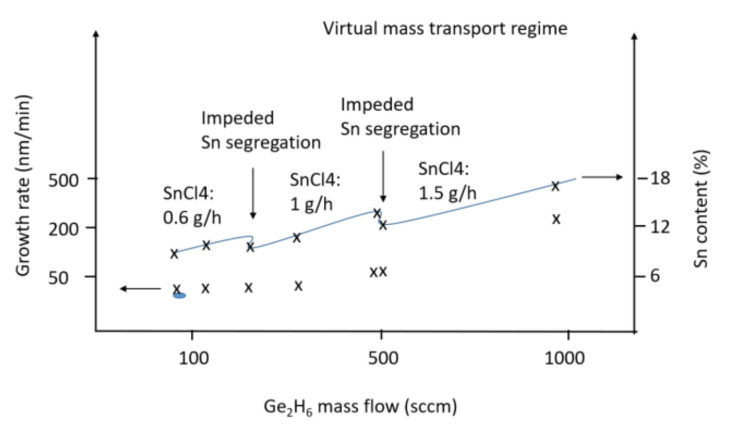
Growth parameters of GeSn deposition and its related Sn contents.

Prior to GeSn growth, a Ge buffer with a thickness of at least 1000 nm is necessary to be deposited for high quality epitaxy. The Ge buffer layer contains two layers which are grown at a temperature of 450 °C for the low layer, and at 650 °C for the cap layer. The first layer is highly defective; meanwhile, the second layer has significantly higher epitaxial quality. An annealing treatment at 850 °C ensures the defect density is minimized to a level of 10^7^ cm^−2^. Since the main goal of this study is to incorporate high Sn content in Ge, the quality of the buffer layer is very important. Figure 3 illustrates the series of grown samples in this study. The amount of SnCl_4_ introduced into the epi chamber was increased in three steps of 0.8, 1, and 1.5 g/h. In these samples, Sn segregation appears as Sn dots on the surface of the GeSn layer. The sizes of the Sn dots depend on the thickness of the GeSn layer, and they become larger for thicker GeSn layers. Therefore, it is important in the characterization of GeSn layers that the presence of Sn dots not be misjudged as surface roughness, especially when they are small for thin GeSn layers. Since Sn atoms are large, and since the heat of sublimation for Sn is less than that for Ge, segregation of Sn in Ge buffer occurs from both chemical and size effects. 

In general, the growth of Si-based materials using CVD follows two regimes: kinetic and mass transport. In kinetic mode, the growth rate is temperature-dependent, whereas in mass transport mode the growth rate is dependent on reactant gas partial pressures. In this study, GeSn growth occurs at 280–310 °C; then, it is expected that epitaxy follows the kinetic mode. As we know, Sn segregation depends strongly on the introduced Sn content; meanwhile, Figure 3 illustrates that Sn segregation is decreased by increasing the Ge partial pressure, and diminishes when Ge partial pressure reaches a critical value. Surprisingly, the growth rate is also increased by increasing the Ge partial pressure. By applying high Ge partial pressure, the number of Ge atoms is increased, resulting in a dramatic change to the kinetics of atoms where lateral diffusion of atoms becomes limited. We believe Sn segregation is decreased as a result of the high growth rate and limited lateral diffusion of Sn atoms. This condition of growth could be called virtual mass transport mode at low temperature growth. Reaching this point is the key issue for significant corporation of Sn into a Ge crystal matrix to occur. In the next step, by introducing more Sn atoms, a new condition is reached and it becomes necessary to deal with the excess of Sn atoms during GeSn growth; therefore, Ge atoms will be needed to decrease segregation. By stepwise introduction of more Sn atoms, the Ge partial pressure must be increased with each new step. In Figure 3, three points reflect the optimized growth parameters. As a result of this stepwise approach, 18% of Sn could be incorporated in Ge.

**Figure 4:**
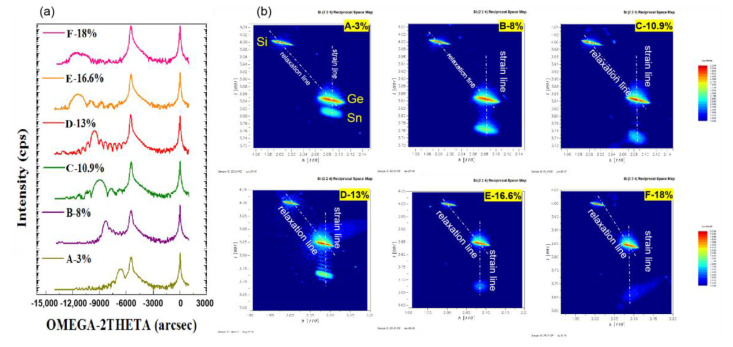
X-ray analysis of GeSn/Ge layers (**a**) rocking curves and (**b**) 224 HRRLMs.

In order to examine the quality of the GeSn layers, high-resolution X-ray diffraction (HRXRD) including reciprocal lattice maps were performed, as shown in Figure 4. The rocking curves illustrate thickness fringes, indicating high interfacial quality. Layer quality is often estimated using the full-width half-maximum (FWHM) method, but in these samples the strain relaxation was not the same due to different layer thicknesses. Therefore, HRRLM was performed, as shown in Figure 4b, to determine strain in both vertical and lateral growth directions. In this way, the strain relaxation could be measured and discussed. HRRLM was performed around (2 2 4) reflection, which is most sensitive to the defects. This is due to the low incident angle of the X-ray beam at 8.7° which makes a long propagation of the X-ray beam along the GeSn layer, and easily reveals the defects [52]. The positions of GeSn and Ge peaks in the reciprocal lattice were determined, then the mismatch parameters were calculated. In the HRRLMs, the shape of the GeSn peak and the diffused scattering provide information about the presence of Sn dots within the GeSn layer. In these maps, the GeSn peak is aligned with the Ge peak along the (001) direction showing minor strain relaxation. The GeSn peak shifts out but is still along the (001) direction, illustrating the increase in strain in the epilayers. 

For GeSn layers in samples E and F with Sn content of 16.6% and 18%, respectively, low intensity color contours are only observed due to the thinness of these layers. Therefore, the acquisition of X-rays become weak and we need to increase the incoming beam intensity for thin layers. Meanwhile for sample F, there is a scattering intensity around the GeSn peak, which could be related to minor strain relaxation. In conclusion, GeSn layers which are fully strained show good quality in HRRLMs. 

The lattice distortion in Ge due to Sn atoms has to be carefully determined in order to derive Sn composition. The following approach shows how this task was performed by using misfit parameters in the GeSn layers [50,51,52]. The misfit parameters were calculated through reading data from the HRRLM of 2*θ* for substrate and the epilayer:

(1) $$f_{{}_{⊥}}=\frac{\Delta a_{z}}{a_{sub}}=\frac{a_{lay}^{z}-a_{sub}}{a_{sub}}=\frac{\sin\theta_{sub}\cos(\omega_{sub}-\theta_{sub})}{\sin\theta_{lay}\cos(\omega_{lay}-\theta_{lay})}-1$$

(2) $$f_{x,y}=\frac{\Delta a_{x,y}}{a_{sub}}=\frac{a_{lay}^{x,y}-a_{sub}}{a_{sub}}=\frac{\sin\theta_{sub}\sin(\omega_{sub}-\theta_{sub})}{\sin\theta_{lay}\sin(\omega_{lay}-\theta_{lay})}-1$$

The total misfit was determined from the following relationship:

(3) $$f=(f_{z}-f_{x,y})\frac{1-v}{1+v}+f_{x,y}$$

where *ν* stands for the Poisson ratio for the material. A serious problem which arose was determining the *ν* value for the GeSn material; in general, this value can be written in terms of elastic constants, *Cij* (see Table 2).

(4) $$\nu =\frac{c_{12}}{c_{12}+c_{11}}$$

As an example, the elastic constant *Cij*-value for a certain alloy composition *Ge*_1-x_*Sn*_x_ can be obtained from Vegard’s law, shown as follows:

(5) $$cij(Ge_{1-x}Sn_{x})=(1-x)cij(Ge)+xcij(Sn)$$

It is important to mention here that Equation (5) provides an approximation to calculate *Cij* values, and this will affect the Poisson ratio value and Sn composition (in the range of this study). Later, the validity of our data were compared with RBS data. The strain relaxation (*R*) is an important value which indicates how much strain has been released. Relaxation is expressed in percentage, and can be obtained from the following:

(6) $$R=\frac{f_{x,y}}{f}$$

For alloy materials, the composition is commonly determined by applying Vegard’s law:

(7) $$f=\frac{x\times a_{B}}{a_{A}}$$

The Poisson ratio is obtained from the corresponding *Cijs* according to Equation (4), then the lattice constant for *GeSn* can be determined. The Sn content extracted with high precision can be derived from the lattice constant for a composition, according to the following equation [50,51]:

(8) $$a_{GeSn}(x)=a_{Sn}x+\theta_{GeSn}x(1-x)+a_{Ge}(1-x)$$

where *θ_GeSn_* is a constant which relates to *GeSn* alloying and is 0.166 Å for *x* ≤ 0.20 [47,48]. The calculated *Sn* contents for *GeSn* layers from HRRLMs are illustrated in Table 3.

**Figure 5:**
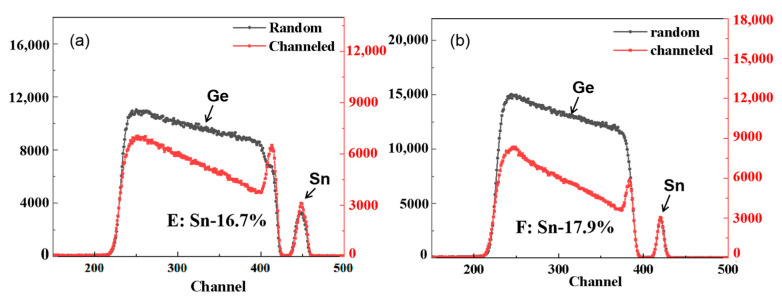
RBS spectra of sample E and sample F.

In order to ensure the accuracy of Sn content in GeSn layers, RBS analysis was performed. The results of samples E and F are shown in Figure 5. The obtained values were 18 and 16.6, respectively, which were consistent with XRD data. This also indicates that the approximation method used to find *Cij* values with Equation (5) was valid. 

**Figure 6:**
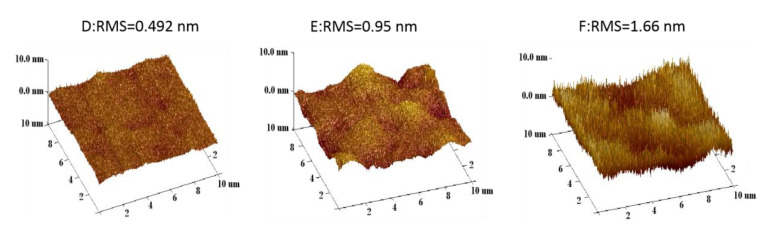
AFM analysis of samples D, E, and F.

The surface roughness of GeSn layers is also a very important consideration for devices, especially in the multilayers. AFM results of the GeSn samples performed are illustrated in Figure 6. The images show that the surface roughness of the GeSn layers have RMS values in the range of 0.492 to 1.66 nm for sample D to sample F, respectively. Sample D has the lowest roughness. These samples were also observed by optical microscope with Normanski filters (not shown here), and the surface roughness was best for sample D. This can also be verified by regular diffraction peaks in HRXRD that show sample D has the best crystal quality. Thus, the surface of sample D is the smoothest.

**Figure 7:**
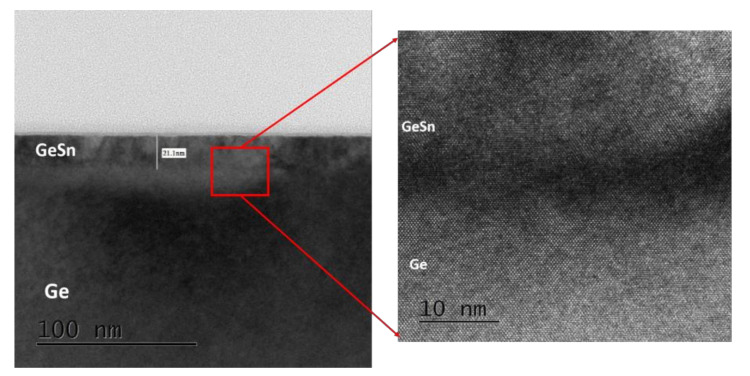
HRTEM cross-sectional images of Ge_0.82_Sn_0.18_ (sample F).

In this series, sample F was analyzed by HRTEM, as shown in Figure 7. The figure reveals that there is a clear and steep interface distribution between Ge and GeSn, and that the atoms of Ge and GeSn are arranged very neatly. This symbolizes that film of GeSn sample used in this investigation had high epitaxial quality [53]. Therefore, for this sample, the greater surface roughness shown in Figure 6 may relate to minor strain relaxation.

### 3.2. Strain Modulation by Lateral Etching

The growth of GeSn/Ge (buffer) yields compressively strained GeSn layers; for direct bandgap transition, the strain has to be modulated. There are three ways to achieve strain relaxation of GeSn layers: I, GeSn layers are grown in a meta-stable region, where the strain modulation then depends on layer thickness and growth temperature; II, by gradually etching the Ge buffer layer so that the GeSn layer partially relaxes; and III, by post annealing the GeSn layer. The third option results in precipitation of Sn atoms in the Ge matrix, so this is not an appropriate method for device applications.

**Figure 8:**
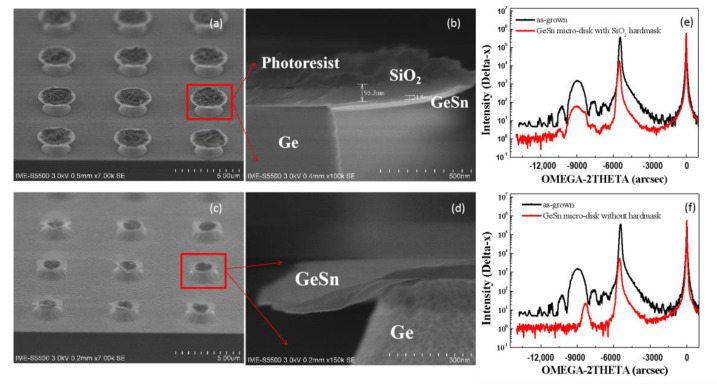
(**a**,**b**) SEM of GeSn micro-disk with SiO_2_ hard mask; (**c**,**d**) SEM of GeSn micro-disk without hard mask; HRXRD rocking curve of micro-disks for samples (**e**) with SiO_2_ hard mask and (**f**) without hard mask; black-colored spectra represent GeSn before etching, and red spectra represent GeSn after etching.

In our experiments, by a vertical etching of GeSn/Ge and later, a lateral etching of Ge, micro-disks could be formed. Figure 8a–f shows how strain in GeSn could be affected by selective etching of Ge to the GeSn layer in a lateral direction. In these experiments, etching of the Ge layer was performed with/without an SiO_2_ hard mask. In both cases, Ge could be successfully etched without any damage to the GeSn layer. However, the GeSn layer bent at the edge for the case without SiO_2_ compared to the case with SiO_2_, as shown in Figure 8c,d, since there was no SiO_2_ layer that could hold the layer and delay or affect strain relaxation. 

The HRXRD analysis from these samples from before and after etching in Figure 8e,f confirm that there is a clear shift of the GeSn peak towards the Ge peak for micro-disk samples without the SiO_2_ hard mask. There are no interference peaks in Figure 8f which indicate that the GeSn thin interface or surface were affected as a result of partial strain relaxation. 

**Figure 9:**
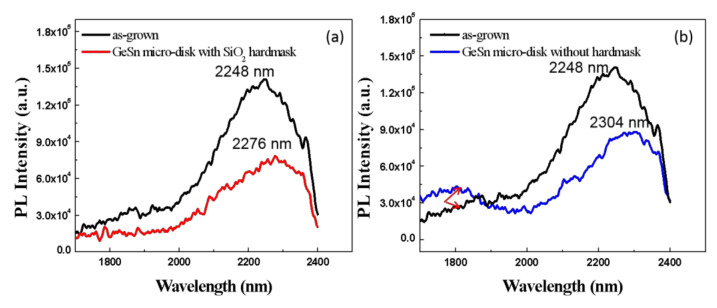
(**a**) PL of GeSn before etching (black line) and GeSn micro-disk with SiO_2_ as hard mask (red line); (**b**) PL of GeSn as-grown (black line) and GeSn micro-disk without hard mask.

As a result of the partial relaxation of the film, the influence of strain becomes weaker where the Γ energy valley shifts downward, and the bandgap of the material becomes smaller. Theoretically, this results in red-shift in the PL spectrum [39]. Figure 9a shows the PL test map for as-grown micro-disks, and micro-disks with SiO_2_ hard mask at room temperature. The figure shows that the GeSn peak at 2248 nm red-shifts to 2276 nm after micro-disk formation. Meanwhile, in Figure 9b the red-shift of the sample without SiO_2_ is from 2248 nm to 2304 nm, which is almost double the shift of the sample in Figure 9a. The amount of red-shift in these samples is related to strain relaxation, which is consistent with HRXRD results.

### 3.3. Strain Modulation of GeSnOI by Vertical Etching

**Figure 10:**
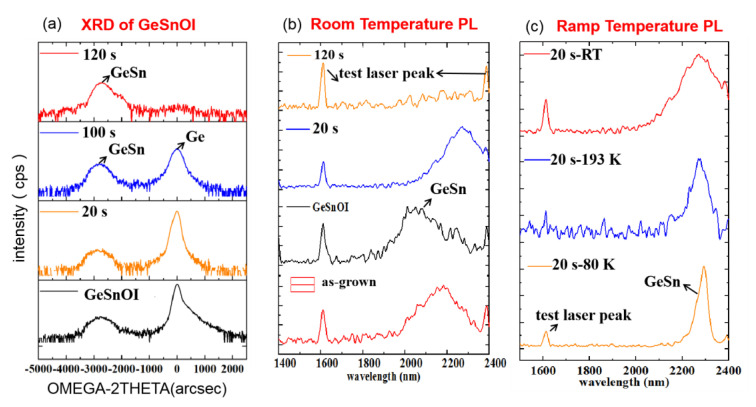
(**a**) XRD of GeSnOI as-grown (black line) and after wet etchings of 20 s (orange line), 100 s (blue line), and 120 s (red line); (**b**) PL at room temperature of GeSn as-grown (red line), GeSnOI (black line), and after wet etchings of 20 s (blue line), 100 s (amaranth line), and 120 s (orange line); and (**c**) PL of GeSnOI after wet etching of 20 s at temperatures of 80 K (orange line), 193 K (blue line), and room temperature (red line).

The strain modulation of GeSnOI was studied by stepwise etching the Ge buffer. Figure 10 shows HRXRD results of as-grown and after 20 s, 100 s, and 120 s etching times of GeSnOI. In these experiments, the intensity of the Ge peak decreases gradually with increases in etching time, and later appears as Ge is completely etched away. 

Figure 10b shows the GeSn peak has a blue-shift after the bonding process. It is believed that this shift is a result of the annealing treatment (300 °C 1 h) which could cause a number of Sn atoms to be pushed out from substitutional sites, resulting in strain reduction. This explanation originates from the fact that we observed no defects in HRTEM results. Meanwhile, the etching of the Ge buffer creates a red-shift, as expected. The red-shift is caused by stress release and bandgap alignment in GeSn. Table 4 and Figure 10c demonstrate more PL investigation on the GeSnOI sample with a 20-s etching of Ge buffer at different temperatures. The PL peak’s FWHM at room temperature (shown as Table 4) is decreased at low temperature analysis, which is consistent with freezing of imperfections in the epilayer.

**Figure 11:**
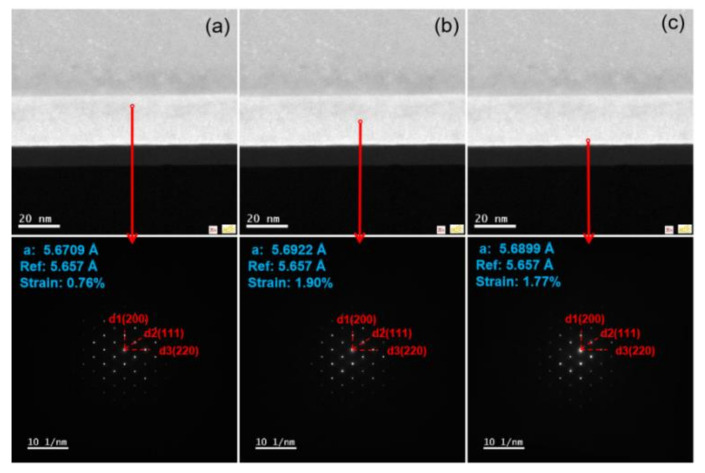
NBD in different GeSn regions of sample wet etched for 20 s; the reference is bulk Ge with lattice constant 5.657 Å. (**a**) Up; (**b**) middle; (**c**) down.

In order to find out the strain distribution in the above sample GeSnOI, NBD analysis was performed, as shown in Figure 11a–c, and more details about estimated strain values are demonstrated in Table 5. The strain is distributed at the top level in the middle of the GeSn layer and more relaxation occurs in the vertical direction close to the Ge buffer. No defects are created in GeSn layers, and by removing more Ge buffer the strain is constantly decreased.

## 4. Conclusions

In this research, a novel growth method was proposed to incorporate Sn content up to 18% in a Ge matrix. Furthermore, we propose that using XRD reciprocal maps is an accurate method to determine Sn content in GeSn layers instead of the time-consuming and expensive conventional RBS analysis. GeSn/Ge were patterned to form micro-disks where the Ge buffer was etched using a selective wet etch. The strain was modulated by removing the Ge buffer where PL and XRD were applied to study the strain relaxation. Results showed that when no (SiO_2_) hard mask was used for micro-disk formation, strain relaxation occurred only when the micro-disk was bending. GeSnOI was also formed and the strain in GeSn could be modulated by vertical etching of the Ge buffer, causing red-shift in the PL spectrum. All samples showed PL at room temperature. This study provides vital information for the synthesis of GeSn layers with high Sn content, and for using these layers in advanced photonic applications within the SWIR spectral region.

## Figures and Tables

**Table 1:** Process details of vertical wet etching of Ge.

Sample	GeSn Peak Position /Arceconds	Ge before Etching/nm	Etching Time/s	Ge after Etching/nm	Etching Rate nm/s
GeSnOI	NH_4_OH:H_2_O_2_:H_2_O (1:4:25)	710	0	710	0
20 s	NH_4_OH:H_2_O_2_:H_2_O (1:4:25)	710	20	500	10.55
100 s	NH_4_OH:H_2_O_2_:H_2_O (1:4:25)	710	100	100	6.11
120 s	NH_4_OH:H_2_O_2_:H_2_O (1:4:25)	710	120	0	5.925

**Table 2:** Elastic constants of the group IV elements [50,51].

Elastic Constant	Ge	Sn	Si	C
c_11_ (Mbar)	1.26	0.69	1.67	10.79
c_1__2_ (Mbar)	0.44	0.29	0.65	1.24

**Table 3:** Sn content in *GeSn* layers obtained from HRRLMs.

Sample	GeSn Peak Position /Arceconds	Mismatch /ppm	ɑ_⊥_/Å	x
A	−6590	5184	5.70925	0.036
B	−8330	12,328	5.78025	0.084
C	−8990	15,554	5.81246	0.106
D	−9560	18,139	5.83832	0.124
E	−11,340	25,734	5.91466	0.175
F	−11,267	25,548	5.91278	0.176

**Table 4:** PL data for the GeSn peak at 80 K and at room temperature (RT) after Ge vertical wet etching.

Sample	FWHM-80 K/nm	Peak of GeSn-80 K/nm	FWHM-RT/nm	Peak of GeSn-RT/nm
GeSn Before bonding	--	--	251	2166
GeSnOI	129	2040	134	2039
20 s	60	2286	284	2270
120 s	26	2279	345	2232

**Table 5:** NBD results about GeSnOI strain before etching, and after wet etching for 20 s and 120 s.

Sample	Bulk Strain	Strain_⊥_(002)	Strain_//_(220)
GeSnOI	2.79%	2.11%	0.47%
20 s	1.90%	1.60 %	0.17 %
120 s	1.19%	2.24%	−0.32%

## Data Availability

The data presented in this study are available on request from the corresponding authors.

## References

[1] Moontragoon P., Ikonić Z., Harrison P. (2007). Band structure calculations of Si–Ge–Sn alloys: Achieving direct band gap materials. Semicond. Sci. Technol..

[2] Radamson H.H., Radamson H.H., Luo J., Simoen E., Zhao C. (2018). 3—Strain Engineering. CMOS Past, Present and Future.

[3] Lei D., Lee K.H., Bao S., Wang W., Masudy-Panah S., Yadav S., Kumar A., Dong Y., Kang Y., Xu S. The first GeSn FinFET on a novel GeSnOI substrate achieving lowest S of 79 mV/decade and record high Gm, int of 807 μS/μm for GeSn P-FETs. Proceedings of the 2017 Symposium on VLSI Technology.

[4] Radamson H.H., Zhu H.L., Wu Z.H., He X.B., Lin H.X., Liu J.B., Xiang J.J., Kong Z.Z., Wang G.L. (2020). State of the Art and Future Perspectives in Advanced CMOS Technology. Nanomaterials.

[5] Koliopoulou S., Dimitrakis P., Goustouridis D., Normand P., Pearson C., Petty M.C., Radamson H., Tsoukalas D. (2006). Metal nano-floating gate memory devices fabricated at low temperature. Microelectron. Eng..

[6] Liu L., Liang R., Wang G., Radamson H.H., Wang J., Xu J. Investigation on direct-gap GeSn alloys for high-performance tunneling field-effect transistor applications. Proceedings of the 2017 IEEE Electron Devices Technology and Manufacturing Conference (EDTM).

[7] Miao Y., Wang G., Kong Z., Xu B., Zhao X., Luo X., Lin H., Dong Y., Lu B., Dong L. (2021). Review of Si-Based GeSn CVD Growth and Optoelectronic Applications. Nanomaterials.

[8] Li Y., Wang G., Akbari-Saatlu M., Procek M., Radamson H.H. (2021). Si and SiGe Nanowire for Micro-Thermoelectric Generator: A Review of the Current State of the Art. Front. Mater..

[9] Noroozi M., Hamawandi B., Toprak M.S., Radamson H.H. Fabrication and thermoelectric characterization of GeSn nanowires. Proceedings of the 2014 15th International Conference on Ultimate Integration on Silicon (ULIS).

[10] Gurdal O., Desjardins P., Carlsson J.R.A., Taylor N., Radamson H.H., Sundgren J.E., Greene J.E. (1998). Low-temperature growth and critical epitaxial thicknesses of fully strained metastable Ge_1−x_Sn_x_ (x ≤ 0.26) alloys on Ge (001) 2 × 1. J. Appl. Phys..

[11] Ni W.X., Ekberg J.O., Joelsson K.B., Radamson H.H., Henry A., Shen G.D., Hansson G.V. (1995). A silicon molecular beam epitaxy system dedicated to device-oriented material research. J. Cryst. Growth.

[12] Rathore J., Nanwani A., Mukherjee S., Das S., Moutanabbir O., Mahapatra S. (2021). Composition uniformity and large degree of strain relaxation in MBE-grown thick GeSn epitaxial layers, containing 16% Sn. J. Phys. D Appl. Phys..

[13] Al-Kabi S., Ghetmiri S.A., Margetis J., Du W., Mosleh A., Dou W., Sun G., Soref R.A., Tolle J., Li B. (2016). Study of High-Quality GeSn Alloys Grown by Chemical Vapor Deposition towards Mid-Infrared Applications. J. Electron. Mater..

[14] Vincent B., Gencarelli F., Bender H., Merckling C., Douhard B., Petersen D.H., Hansen O., Henrichsen H., Meersschaut J., Caymax M. (2011). Undoped and in-situ B doped GeSn epitaxial growth on Ge by atmospheric pressure-chemical vapor deposition. Appl. Phys. Lett..

[15] Margetis J., Mosleh A., Al-Kabi S., Ghetmiri S.A., Du W., Dou W., Benamara M., Li B., Mortazavi M., Naseem H.A. (2017). Study of low-defect and strain-relaxed GeSn growth via reduced pressure CVD in H_2_ and N_2_ carrier gas. J. Cryst. Growth.

[16] Wirths S., Buca D., Mussler G., Tiedemann A.T., Holländer B., Bernardy P., Stoica T., Grützmacher D., Mantl S. (2013). Reduced Pressure CVD Growth of Ge and Ge_1−x_Sn_x_ Alloys. ECS J. Solid State Sci. Technol..

[17] Margetis J., Ghetmiri S.A., Du W., Conley B.R., Mosleh A., Soref R., Yu S., Tolle J. (2014). Growth and characterization of epitaxial Ge_1-X_Sn_x_ alloys and heterostructures using a commercial CVD system. ECS Trans..

[18] Radamson H.H., Noroozi M., Jamshidi A., Thompson P.E., Östling M. (2013). Strain engineering in GeSnSi materials. ECS Trans..

[19] Jamshidi A., Noroozi M., Moeen M., Hallén A., Hamawandi B., Lu J., Hultman L., Östling M., Radamson H. (2013). Growth of GeSnSiC layers for photonic applications. Surf. Coat. Technol..

[20] Noroozi M., Abedin A., Moeen M., Östling M., Radamson H.H. (2014). CVD growth of GeSnSiC alloys using disilane, digermane, Tin Tetrachloride and methylsilane. ECS Trans..

[21] Margetis J., Mosleh A., Ghetmiri S.A., Al-Kabi S., Dou W., Du W., Bhargava N., Yu S.-Q., Profijt H., Kohen D. (2017). Fundamentals of Ge_1−x_Sn_x_ and Si_y_Ge_1−x-y_Sn_x_ RPCVD epitaxy. Mater. Sci. Semicond. Processing.

[22] Bertrand M., Casiez L., Quintero A., Chrétien J., Pauc N., Thai Q.M., Khazaka R., Rodriguez P., Hartmann J.M., Chelnokov A. (2020). Reboud, Vertical GeSn electro-absorption modulators grown on Silicon for the mid-infrared. 2020 IEEE Photonics Conference (IPC).

[23] Grant P.C., Dou W., Alharthi B., Grant J.M., Tran H., Abernathy G., Mosleh A., Du W., Li B., Mortazavi M. (2019). UHV-CVD growth of high quality GeSn using SnCl4: From material growth development to prototype devices. Opt. Mater. Express.

[24] Dou W., Alharthi B., Grant P.C., Grant J.M., Mosleh A., Tran H., Du W., Mortazavi M., Li B., Naseem H. (2018). Crystalline GeSn growth by plasma enhanced chemical vapor deposition. Opt. Mater. Express.

[25] Yang J., Hu H., Miao Y., Dong L., Wang B., Wang W., Xuan R. (2019). High-quality GeSn Layer with Sn Composition up to 7% Grown by Low-temperature Magnetron Sputtering for Optoelectronic Application. Materials.

[26] Zheng J., Liu Z., Zhang Y., Zuo Y., Li C., Xue C., Cheng B., Wang Q. (2018). Growth of high-Sn content (28%) GeSn alloy films by sputtering epitaxy. J. Cryst. Growth.

[27] Tolle J., Roucka R., D’Costa V., Menendez J., Chizmeshya A., Kouvetakis J. (2005). Sn-based Group-IV Semiconductors on Si: New Infrared Materials and New Templates for Mismatched Epitaxy. MRS Online Proc. Lib..

[28] Kouvetakis J., Chizmeshya A. (2007). New classes of Si-based photonic materials and device architectures via designer molecular routes. J. Mater. Chem..

[29] Aubin J., Hartmann J.M., Gassenq A., Milord L., Pauc N., Reboud V., Calvo V. (2017). Impact of thickness on the structural properties of high tin content GeSn layers. J. Cryst. Growth.

[30] Loo R., Shimura Y., Ike S., Vohra A., Stoica T., Stange D., Buca D., Kohen D., Margetis J., Tolle J. (2018). Epitaxial GeSn: Impact of process conditions on material quality. Semicond. Sci. Technol..

[31] Ghetmiri S.A., Du W., Margetis J., Mosleh A., Cousar L., Conley B.R., Domulevicz L., Nazzal A., Sun G., Soref R.A. (2014). Direct-bandgap GeSn grown on silicon with 2230 nm photoluminescence. Appl. Phys. Lett..

[32] Wirths S., Geiger R., von den Driesch N., Mussler G., Stoica T., Mantl S., Ikonic Z., Luysberg M., Chiussi S., Hartmann J.M. (2015). Lasing in direct-bandgap GeSn alloy grown on Si. Nat. Photonics.

[33] Stange D., Wirths S., Geiger R., Schulte-Braucks C., Marzban B., von den Driesch N., Mussler G., Zabel T., Stoica T., Hartmann J.-M. (2016). Optically Pumped GeSn Microdisk Lasers on Si. ACS Photonics.

[34] Thai Q.M., Pauc N., Aubin J., Bertrand M., Chrétien J., Chelnokov A., Hartmann J.M., Reboud V., Calvo V. (2018). 2D hexagonal photonic crystal GeSn laser with 16% Sn content. Appl. Phys. Lett..

[35] Thai Q.M., Chretien J., Bertrand M., Aubin J., Casiez L., Chelnokov A., Hartmann J.-M., Reboud V., Pauc N., Calvo V. (2022). Progress in Germanium Tin (GeSn) Photonic Crystal Lasers. IEEE J. Sel. Top. Quantum Electron..

[36] Chrétien J., Pauc N., Armand Pilon F., Bertrand M., Thai Q.-M., Casiez L., Bernier N., Dansas H., Gergaud P., Delamadeleine E. (2019). GeSn Lasers Covering a Wide Wavelength Range Thanks to Uniaxial Tensile Strain. ACS Photonics.

[37] Joo H.-J., Kim Y., Burt D., Jung Y., Zhang L., Chen M., Parluhutan S.J., Kang D.-H., Lee C., Assali S. (2021). 1D photonic crystal direct bandgap GeSn-on-insulator laser. Appl. Phys. Lett..

[38] Margetis J., Zhou Y., Dou W., Grant P.C., Alharthi B., Du W., Wadsworth A., Guo Q., Tran H., Ojo S. (2018). All group-IV SiGeSn/GeSn/SiGeSn QW laser on Si operating up to 90 K. Appl. Phys. Lett..

[39] Elbaz A., Buca D., von den Driesch N., Pantzas K., Patriarche G., Zerounian N., Herth E., Checoury X., Sauvage S., Sagnes I. (2020). Ultra-low-threshold continuous-wave and pulsed lasing in tensile-strained GeSn alloys. Nat. Photonics.

[40] Elbaz A., Arefin R., Sakat E., Wang B., Herth E., Patriarche G., Foti A., Ossikovski R., Sauvage S., Checoury X. (2020). Reduced Lasing Thresholds in GeSn Microdisk Cavities with Defect Management of the Optically Active Region. ACS Photonics.

[41] Zhou Y., Miao Y., Ojo S., Tran H., Abernathy G., Grant J.M., Amoah S., Salamo G., Du W., Liu J. (2020). Electrically injected GeSn lasers on Si operating up to 100 K. Optica.

[42] Zhou Y., Ojo S., Wu C.-W., Miao Y., Tran H., Grant J.M., Abernathy G., Amoah S., Bass J., Salamo G. (2021). Electrically injected GeSn lasers with peak wavelength up to 2.7 μm. Photonics Res..

[43] Du W., Thai Q.M., Chrétien J., Bertrand M., Casiez L., Zhou Y., Margetis J., Pauc N., Chelnokov A., Reboud V. (2019). Study of Si-Based GeSn Optically Pumped Lasers with Micro-Disk and Ridge Waveguide Structures. Front. Phys..

[44] Reboud V., Gassenq A., Pauc N., Aubin J., Milord L., Thai Q.M., Bertrand M., Guilloy K., Rouchon D., Rothman J. (2017). Optically pumped GeSn micro-disks with 16% Sn lasing at 3.1 μm up to 180 K. Appl. Phys. Lett..

[45] Thai Q.M., Pauc N., Aubin J., Bertrand M., Chretien J., Delaye V., Chelnokov A., Hartmann J.M., Reboud V., Calvo V. (2018). GeSn heterostructure micro-disk laser operating at 230 K. Opt. Express.

[46] Senaratne C.L., Gallagher J.D., Jiang L., Aoki T., Smith D.J., Menéndez J., Kouvetakis J. (2014). Ge_1-y_Sn_y_ (y = 0.01−0.10) alloys on Ge-buffered Si: Synthesis, microstructure, and optical properties. J. Appl. Phys..

[47] Fischer A.C., Belova L.M., Rikers Y.G.M., Malm B.G., Radamson H.H., Kolahdouz M., Gylfason K.B., Stemme G., Niklaus F. (2012). 3D Free-Form Patterning of Silicon by Ion Implantation, Silicon Deposition, and Selective Silicon Etching. Adv. Funct. Mater..

[48] Han Y., Li Y., Song Y., Chi C., Zhang Z., Liu J., Zhu Z., Wang S. (2018). A comparative study of selective dry and wet etching of germanium–tin (Ge_1−x_Sn_x_) on germanium. Semicond. Sci. Technol..

[49] Campo A., Cardinaud C., Turban G. (1995). Comparison of etching processes of silicon and germanium in SF_6_–O_2_ radio-frequency plasma. J. Vac. Sci. Technol. B Microelectron. Nanometer Struct..

[50] Radamson H.H., Thylén L., Radamson H.H., Thylén L. (2015). Chapter 4—Moore’s Law for Photonics and Electronics. Monolithic Nanoscale Photonics–Electronics Integration in Silicon and Other Group IV Elements.

[51] Hansson G.V., Radamsson H.H., Ni W.X. (1995). Strain and relaxation in Si-MBE structures studied by reciprocal space mapping using high resolution X-ray diffraction. J. Mater. Sci. Mater. Electron..

[52] Radamson H.H., Hållstedt J. (2005). Application of high-resolution x-ray diffraction for detecting defects in SiGe(C) materials. J. Phys. Condens. Matter.

[53] Radamson H.H., Joelsson K.B., Ni W.-X., Hultman L., Hansson G.V. (1995). Characterization of highly boron-doped Si, Si_1−x_Ge_x_ and Ge layers by high-resolution transmission electron microscopy. J. Cryst. Growth.

